# Advancing chemical mixture toxicity assessment using advanced in vitro NAMs: current status and future perspectives

**DOI:** 10.3389/ftox.2026.1757928

**Published:** 2026-05-29

**Authors:** Hyunwoo Kim, Donghyeon Kim, Jinhee Choi

**Affiliations:** Department of Environmental Engineering, University of Seoul, Seoul, Republic of Korea

**Keywords:** mixture toxicity, new approach methodologies, advanced *in vitro* models, HTS/HCI, 3D tissue models, exposure route-relevant models, omics technologies

## Abstract

Chemical exposure in real-world settings occurs predominantly as mixtures, whereas toxicological assessment has long been shaped by single-substance paradigms. As interest in more human-relevant and mechanistically informative testing strategies has grown, new approach methodologies (NAMs) have been increasingly incorporated into mixture toxicity research. However, the role of advanced *in vitro* NAMs in chemical mixture toxicity assessment has not yet been examined in a structured manner. This review therefore evaluated how advanced *in vitro* NAMs are currently being applied in mixture toxicity assessment and considered their potential value for regulatory translation. A PubMed-based literature search covering studies published between 2021 and 2025 identified 353 *in vitro* NAM-based studies related to chemical mixture toxicity, of which 70 were classified as advanced *in vitro* NAM-based studies and included for in-depth analysis. Among the advanced *in vitro* NAM categories, route-of-exposure-relevant models were the most frequently applied, followed by Omics, 3D models, stem cell-based models, and HTS/HCI approaches. The reviewed studies addressed diverse mixtures, including air pollutants, PFAS, phthalates, pesticides/herbicides, consumer product-related mixtures, and other environmentally relevant chemical combinations. Across these studies, advanced *in vitro* NAMs were used not only to assess overall mixture effects, but also to compare observed and predicted responses, distinguish mixture effects from those of individual chemicals, identify effect-driving components, and interpret non-additive responses under biologically relevant conditions. The reviewed evidence indicates that advanced *in vitro* NAMs are being used not simply as alternative test systems, but as tools to address key challenges intrinsic to mixture toxicity assessment, including defining relevant mixture entities, identifying toxicity drivers, interpreting non-additivity, and supporting prioritization under combinatorial complexity. These findings suggest that the future value of advanced *in vitro* NAMs in mixture assessment will depend not only on continued technological development, but also on their integration into question-driven and fit-for-purpose testing strategies. From this perspective, advanced *in vitro* NAMs may contribute most effectively when used to generate decision-relevant evidence aligned with specific mixture assessment needs and regulatory contexts.

## 1 Introduction

New approach methodologies (NAMs) have become increasingly important in contemporary toxicology as more human-relevant, mechanistically informative, and fit-for-purpose approaches are sought for chemical safety evaluation (Magurany et al., 2023; Mathisen et al., 2025). In particular, NAMs have expanded the ability to investigate biological responses beyond traditional low-content toxicity testing by enabling higher-throughput screening, response-rich phenotyping, systems-level molecular profiling, and more refined *in vitro* modeling. As a result, NAMs are now recognized not only as alternatives to conventional testing strategies, but also as tools for generating mechanistic evidence that can support more integrated toxicological evaluation (Li and Xia, 2020). Within this broader transition, advanced *in vitro* NAMs have developed rapidly in both technical diversity and biological sophistication. Recent *in vitro* platforms increasingly incorporate richer analytical outputs and more biologically relevant model structures, including high-throughput or high-content screening systems, Omics-based approaches, stem cell-based platforms, three-dimensional tissue models, and exposure route-based models that better reflect inhalation or dermal contact scenarios. Collectively, these approaches make it possible to investigate chemical effects across multiple levels of biological organization, from early molecular perturbation to downstream functional and tissue-level responses (Bridgeman et al., 2025; Deepika et al., 2025). At the same time, how these advanced *in vitro* NAMs are being applied in chemical mixture toxicity assessment has not yet been sufficiently examined in a structured way. Unlike single-substance assessment, mixture toxicity assessment is characterized by challenges that are intrinsic to mixtures themselves (Mustafa et al., 2023). Real-life co-exposures are often incompletely characterized, chemically variable, and compositionally dynamic, making it difficult to define the relevant mixture entity and to determine whether observed effects can be adequately represented by a few dominant components (Rager and Rider, 2023). In addition, mixtures may induce non-additive responses such as synergy or antagonism, yet the biological basis of these effects often remains difficult to interpret (Martin et al., 2020). Furthermore, because the number and diversity of real-world co-exposure scenarios are extremely large, comprehensive testing of all relevant mixtures is not feasible in practice. As a result, mixture assessment increasingly requires prioritization, tiered evaluation, and the integration of multiple lines of evidence. In this context, the value of advanced *in vitro* NAMs lies not simply in offering more sophisticated test systems, but in helping address these mixture-specific challenges by supporting the definition of relevant mixtures, the identification of effect-driving components, the mechanistic interpretation of non-additive effects, and more strategic evaluation under combinatorial complexity (Cattaneo et al., 2023; Nikolopoulou et al., 2023).

Against this background, the present review examines how advanced *in vitro* NAMs are being used in chemical mixture toxicity assessment. Using a PubMed-based literature search covering studies published between 2021 and 2025, we identified recent studies applying advanced *in vitro* NAMs to mixture toxicity research. We first classify the major technology categories used in this field, including high-throughput screening/high-content imaging (HTS/HCI), Omics, stem cell-based models, 3D models, and exposure route-based advanced *in vitro* models. We then analyze how these approaches contribute to addressing key challenges in mixture assessment, particularly the definition of relevant real-life mixtures, identification of effect-driving components, mechanistic interpretation of non-additive effects, and prioritization under combinatorial complexity.

## 2 Study characteristics

**FIGURE 1:**
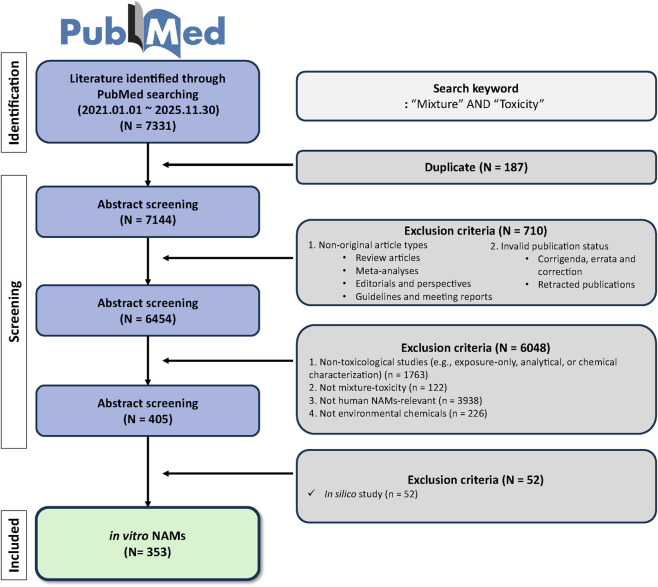
Flow diagram of the literature search and study selection process.

We analyzed publications indexed in the PubMed database to identify recent methodological trends in *vitro* NAMs for chemical mixture toxicity assessment. To ensure broad coverage of the literature, we adopted an intentionally inclusive search strategy using the Boolean search terms [“Mixture” AND “Toxicity”] without restricting the search to specific NAM types or toxicological endpoints. A systematic literature search of studies published between 1 January 2021, and 30 November 2025, initially retrieved 7,331 records. After removal of 187 duplicates, 7,144 records remained for screening. Among these, 710 records were excluded because they were non-original articles or invalid publication types, including review articles, meta-analyses, editorials and perspectives, guidelines and meeting reports, corrigenda, errata, and retracted publications. Consequently, 6,454 records were subjected to title and abstract screening. According to predefined eligibility criteria, studies were further excluded if they were non-toxicological, not focused on mixture toxicity, not relevant to human NAMs, or involved non-environmental chemicals. Following this step, 405 articles were retained for further assessment. Full-text evaluation was then conducted to identify studies specifically addressing *in vitro* NAMs for chemical mixture toxicity assessment, and 52 *in silico* studies were excluded at this stage. Ultimately, 353 studies were included in the final analysis. These studies represent the final body of literature used to characterize recent applications of *in vitro* NAMs in chemical mixture toxicity assessment. The overall screening and study selection process is summarized in Figure 1.

## 3 Research trend of mixture toxicity studies using NAMs

**FIGURE 2:**
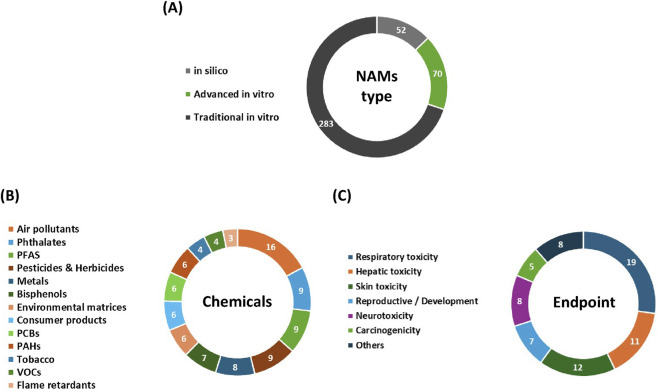
Overview of research trends in mixture toxicity studies using NAMs **(A)** distribution of studies according to NAM type **(B)** distribution of studies by chemical type, and **(C)** distribution of studies by toxicity endpoint. Values indicate the number of studies.

As shown in Figure 2A, the reviewed literature was dominated by traditional *in vitro* studies (282 studies), whereas advanced *in vitro* studies accounted for a smaller but substantial proportion (71 studies). In silico studies represented an additional 52 studies. Because the primary aim of this review was to examine methodological trends in advanced *in vitro* NAMs for mixture toxicity assessment, the subsequent analysis focused on the 71 advanced *in vitro* studies. Regarding chemical groups (Figure 2B), air pollutants were the most frequently investigated category (16 studies). Phthalates and per- and polyfluoroalkyl substances (PFAS) were also commonly represented, with nine studies each, followed by pesticides and herbicides (9 studies), metals (8 studies), and bisphenols (7 studies). Environmental matrices accounted for seven studies, while consumer products and polychlorinated biphenyls (PCBs) were represented in six studies each. Polycyclic aromatic hydrocarbons (PAHs) and tobacco were each investigated in four studies, and volatile organic compounds (VOCs) and flame retardants were less frequently represented, with four and three studies, respectively. Overall, this distribution indicates that current advanced *in vitro* NAM-based mixture toxicity research has been concentrated on environmentally relevant contaminant groups, particularly air pollutants and several major classes of chemicals of regulatory concern. Regarding toxicity endpoints (Figure 2C), respiratory toxicity was the most frequently studied endpoint (19 studies), followed by skin toxicity (12 studies) and hepatic toxicity (11 studies). Reproductive/developmental toxicity and neurotoxicity were each represented in eight studies, whereas carcinogenicity-related endpoints were less frequent (5 studies). The remaining studies were classified as others (8 studies). Taken together, these findings suggest that current advanced *in vitro* NAM-based mixture toxicity studies are primarily focused on organ- and system-specific outcomes, with particular emphasis on respiratory, dermal, and hepatic toxicity.

### 3.1 Targeted chemical mixtures

**FIGURE 3:**
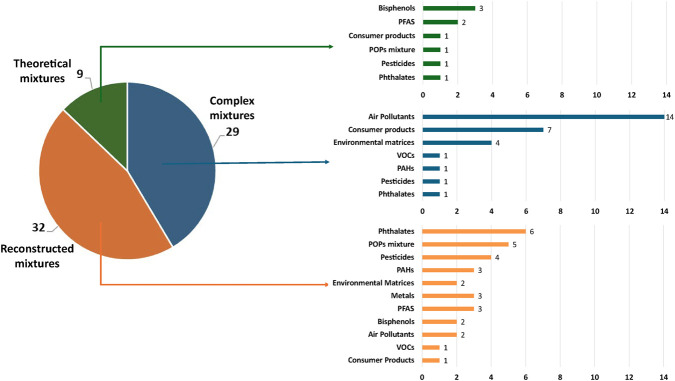
Distribution of mixture design strategies across different chemical categories. Values indicate the number of studies.

The distribution of mixture design strategies across chemical categories was not uniform in the reviewed studies, as summarized in Figure 3. Overall, reconstructed mixtures accounted for the largest proportion (n = 33), followed by complex mixtures (n = 29) and theoretical mixtures (n = 9). This distribution suggests that recent mixture toxicity studies have not been limited to simplified hypothetical combinations, but have increasingly incorporated either experimentally reconstructed mixtures or more realistic exposure entities such as environmental matrices and consumer product-related samples. At the same time, the use of these design strategies varied substantially across chemical categories.

More specifically, theoretical mixtures were represented mainly by bisphenols (n = 3) and PFAS (n = 2), whereas consumer products, POPs mixtures, pesticides, and phthalates were each represented by only one study. This pattern suggests that theoretical mixtures have mainly been used for relatively well-defined chemical groups, often in settings where specific combination hypotheses can be tested under controlled conditions. In contrast, complex mixtures were dominated by air pollutants (n = 14), followed by consumer products (n = 7) and environmental matrices (n = 4), while VOCs, PAHs, pesticides, and phthalates were each represented by one study. This distribution indicates that complex mixture designs have been used primarily for exposure scenarios in which the mixture is treated as a realistic whole entity rather than reduced to a limited number of predefined components. By comparison, reconstructed mixtures were most frequently reported for phthalates (n = 6), followed by POPs mixtures (n = 5) and pesticides (n = 4). PAHs, environmental matrices, metals, and PFAS each accounted for three studies, bisphenols for two, air pollutants for two, and VOCs and consumer products for one study each. This pattern suggests that reconstructed mixtures have been widely used when researchers aim to preserve some exposure relevance while still retaining sufficient control over mixture composition for mechanistic or comparative evaluation.

Taken together, these distributions suggest that recent targeted mixture studies do not appear to follow a single uniform design principle. Rather, different mixture construction logics appear to be applied depending on the characteristics of the chemical group and the exposure context (Table 1). PFAS were often investigated as relatively well-defined combinations of major compounds, using both reconstructed and theoretical mixture designs. Phthalates were more often studied using reconstructed mixtures informed by biomonitoring or other human exposure data, particularly in reproductive or developmental contexts. By contrast, bisphenols were more commonly examined using theoretical designs combining structurally related analogues. Overall, PFAS and phthalates appeared to be more closely linked to exposure-relevant reconstruction, whereas bisphenols showed a stronger tendency toward theory-driven comparison.

**TABLE 1:** Representative studies by chemical group (PFAS, Phthalates, Bisphenols).

Chemical group	Bio model	Toxicity endpoint	Mixture combination	Mixture rationale	Mixture interaction	References
PFAS	MCF-10A	Carcinogenicity	PFOS, PFOA	Reconstructed mixtures: PFAS-relevant exposure pair	Synergistic	Pierozan et al. (2023)
	HepG2	Hepatic toxicity	PFOS, PFOA, PFHxA	Theoretical mixtures: equimolar ternary PFAS	Additive	Carberry et al. (2025)
	hESC-derived cerebral organoids	Neurotoxicity	PFOA, PFOS, PFHxS	Reconstructed mixtures: maternal serum-based PFAS	None (Whole-mixture)	Lu et al. (2024)
	HepG2, HepaRG	Hepatic toxicity	PFOS, PFOA, PFHxS, PFNA, PFDA	Reconstructed mixtures: human exposure-based PFAS	None (Whole-mixture)	Alijagic et al. (2024)
	3D human primary liver microtissues	Hepatic toxicity	PFOA, PFOS, PFNA, PFHxS, PFDA, PFUnA, PFBS *etc.*	Theoretical mixtures: equimolar PFAS complexity series	Additive	Addicks et al. (2023)
Phthalates	HepG2	Hepatic toxicity	DEHP, DBP	Theoretical mixtures: fixed-ratio binary phthalates	Synergistic/antagonistic	Dong et al. (2023)
	Endometrial stromal cell lines and 3D spheroids	DART	MEHHP, MEHP, MEOHP, MECPP	Reconstructed mixtures: biomonitoring	None (Driver identification)	Lavogina et al. (2024)
	Primary human endometrial, T-HESC, Ishikawa cells	DART	MEHHP, MEHP, MEOHP, MECPP	Reconstructed mixtures: biomonitoring	None	Visser et al. (2024)
	Granulosa cell lines, primary ovarian cells	DART	MEHHP, MEHP, MEOHP, MECPP	Reconstructed mixtures: cohort-based phthalates	None	Tarvainen et al. (2023)
	Primary human ovarian stromal cell 3D spheroids	DART	MEHP, MEP, MiBP*etc.*	Reconstructed mixtures: urinary concentration-based	None	Nikanfar et al. (2025)
	Reconstructed human epidermis, HaCaT, THP-1	Skin toxicity	DiPeP isomers	Complex samples: UVCB plasticizer	None (Whole-mixture)	Rodrigues et al. (2023)
Bisphenols	HepG2 3D spheroids	Hepatic toxicity	BPA, BPAP, BPC	Theoretical mixtures: binary bisphenol analogues	Antagonistic	Štampar et al. (2023)
	Adipose-derived stem cells	Adipogenic/obesogenic effects	BPA, BPS, BPF	Theoretical mixtures: equimolar ternary bisphenols	Mixed/non-monotonic	Reina-Pérez et al. (2022)
	Primary mesenchymal stem cells	Adipogenic effects	BPA, BPS, BPF, BPB, BPC, BPAF	Theoretical mixtures: sub-active bisphenol mixture	Additive	Norgren et al. (2022)

Abbreviations: hESC, human embryonic stem cell; DART, developmental and reproductive toxicity; T-HESC, telomerase-immortalized human endometrial stromal cells; UVCB, substances of unknown or variable composition, complex reaction products, or biological materials; PFAS, per- and polyfluoroalkyl substances; PFOS, perfluorooctane sulfonate; PFOA, perfluorooctanoic acid; PFHxA, perfluorohexanoic acid; PFHxS, perfluorohexane sulfonate; PFNA, perfluorononanoic acid; PFDA, perfluorodecanoic acid; PFUnA, perfluoroundecanoic acid; PFBS, perfluorobutane sulfonate; DEHP, di (2-ethylhexyl) phthalate; DBP, dibutyl phthalate; MEHHP, mono (2-ethyl-5-hydroxyhexyl) phthalate; MEHP, mono (2-ethylhexyl) phthalate; MEOHP, mono (2-ethyl-5-oxohexyl) phthalate; MECPP, mono (2-ethyl-5-carboxypentyl) phthalate; MEP, monoethyl phthalate; MiBP, mono-isobutyl phthalate; MBzP, monobenzyl phthalate; MCPP, mono (3-carboxypropyl) phthalate; MBP, monobutyl phthalate; DiPeP, di (pentyl) phthalate; BPA, bisphenol A; BPAP, bisphenol AP; BPC, bisphenol C; BPS, bisphenol S; BPF, bisphenol F; BPB, bisphenol B; BPAF, bisphenol AF.

Differences were also observed in terms of mixture interaction. For PFAS, both additive and synergistic responses were reported, although some studies focused primarily on describing whole-mixture response characteristics rather than formally testing interaction. In the case of phthalates, studies more often emphasized driver identification or comparisons among reconstructed mixtures than explicit quantitative classification of mixture interaction as additive, synergistic, or antagonistic. For bisphenols, additive, antagonistic, and non-monotonic responses were all reported, suggesting that mixture interaction in this group may depend strongly on the endpoint and model system used. These observations may suggest that mixture interaction is influenced not only by the chemical group itself, but also by how the mixture is constructed, which NAM platform is applied, and which endpoints are selected.

Accordingly, recent research on targeted chemical mixtures appears to be moving beyond the simple question of which chemicals are combined, and toward a more structured consideration of why mixtures are constructed in a given way and how they are interpreted analytically. In other words, theoretical mixtures appeared to be more common in groups suited to similarity-driven comparison such as bisphenols, reconstructed mixtures were more evident in groups with relatively rich human exposure information such as phthalates and some PFAS, and complex or whole-mixture approaches were more prominent for air pollutants and product- or extract-related exposure units. This overall pattern may indicate that targeted mixture assessment is becoming increasingly refined in ways that better reflect both the properties of specific chemical groups and the realities of human exposure scenarios. More detailed study-level information and the full list of reviewed articles are summarized in Supplementary Tables S1–S5.

### 3.2 Target endpoints

As shown in Figure 2C, the distribution of endpoints in advanced *in vitro* NAM-based mixture toxicity studies was not evenly spread across toxicological domains, but instead tended to cluster around specific target organs and major exposure routes. Overall, respiratory, dermal, and hepatic toxicity were the most prominent categories, followed by reproductive/developmental toxicity and neurotoxicity. This pattern may suggest that current mixture studies are being developed primarily around organ systems that represent major sites of contact or toxicological concern under environmental and consumer-related exposure scenarios.

Among these, respiratory toxicity was one of the clearest areas in which exposure scenario–based NAMs were actively applied. These studies commonly addressed inhalation-relevant mixtures such as air pollutants, combustion products, e-cigarette aerosols, volatile organic compounds, and particulate matter, often using air–liquid interface systems, co-culture models, or organotypic airway platforms. The endpoints extended beyond general cytotoxicity and frequently included oxidative stress, inflammatory responses, barrier dysfunction, and DNA damage, suggesting a focus on early tissue-relevant responses under more realistic inhalation conditions. A similar tendency was observed for dermal toxicity, where reconstructed epidermis models, full-thickness skin models, and *ex vivo* skin explants were often used to evaluate mixtures related to consumer products or everyday environmental exposure, including fragrances, surfactants, cleaning formulations, textile chemicals, and air pollutants. The main endpoints included skin irritation, sensitization-related responses, cytokine release, barrier dysfunction, and oxidative stress. Together, these studies suggest that dermal endpoint evaluation is increasingly moving beyond simple irritation testing toward more exposure-relevant biological responses. By contrast, hepatic toxicity appeared to represent a major area in which defined mixtures were combined with mechanistic or Omics-based readouts. Well-defined mixtures of PFAS, phthalates, herbicides, PAHs, and bisphenols were frequently assessed in hepatic models such as HepG2, HepaRG, C3A, primary liver microtissues, and liver organoids. Common endpoints included transcriptomic or metabolomic perturbation, lipid metabolism disruption, oxidative stress, ER stress, and mitochondrial dysfunction, indicating that hepatic studies have become one of the main domains for more structured mixture evaluation and mechanistic interpretation. Although represented by fewer studies, reproductive/developmental toxicity and neurotoxicity were notable for their reliance on biologically advanced platforms such as human stem cell-derived systems and 3D organoids. In these categories, endpoints such as differentiation, lineage specification, neurite outgrowth, synaptogenesis, and neurotransmitter release were more common than simple viability-based outcomes, highlighting the value of advanced *in vitro* NAMs for capturing higher-order biological processes.

Overall, the endpoint distribution appears to reflect not only toxicological focus, but also differences in exposure route, target organ, and NAM platform. Respiratory and dermal toxicity were closely linked to exposure-route based models, hepatic toxicity to Omics-based and component-oriented evaluation, and reproductive/developmental and neurotoxicity to stem cell- and organoid-based systems.

## 4 NAMs for mixture assessment

**FIGURE 4:**
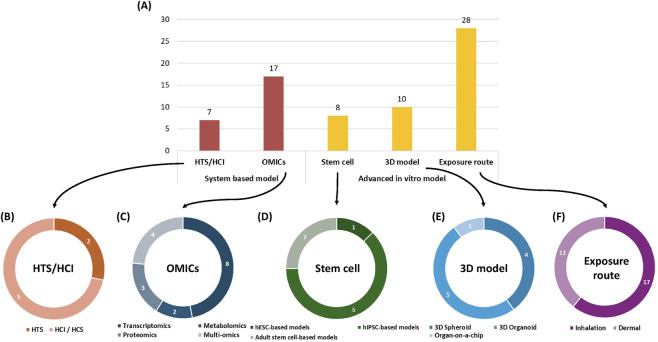
Overview of *in vitro* NAM categories and their subtypes in mixture toxicity studies **(A)** distribution of included studies across the major NAM categories, and subtype distributions within **(B)** HTS/HCI **(C)** Omics **(D)** stem cell-based models **(E)** 3D models, and **(F)** exposure route-based models. Values indicate the number of studies.

To characterize the overall landscape of NAMs applied to mixture toxicity assessment, we categorized the included studies into five major technology groups: HTS/HCI, Omics, stem cell-based models, 3D models, and exposure route-based advanced *in vitro* models. Among these, exposure route-based models were the most frequently applied category (28 studies), followed by Omics (17 studies), 3D models (10 studies), stem cell-based models (9 studies), and HTS/HCI approaches (7 studies). This distribution indicates that recent mixture toxicity studies have increasingly moved beyond conventional high-throughput or single-endpoint screening platforms toward more physiologically relevant systems that better reflect tissue context and realistic exposure conditions (Figure 4A).

The detailed composition of each NAM category is shown in Figures 4B–F. Within the HTS/HCI category, high-content imaging/screening approaches were more commonly used than conventional high-throughput screening assays, with HCI/HCS accounting for five studies and HTS for two. This pattern suggests that image-based phenotypic profiling is increasingly favored for capturing complex cellular responses to mixtures. In the Omics category, transcriptomics was the dominant approach (8 studies), followed by multi-Omics (4 studies), proteomics (3 studies), and metabolomics (2 studies). This distribution suggests that transcriptome-level perturbation analysis remains the most widely adopted systems-level strategy for investigating mixture-induced biological responses, whereas broader multilayer molecular profiling is being incorporated more selectively. Among stem cell-based NAMs, hiPSC-based models were the most frequently used (5 studies), followed by hESC-based systems (2 studies) and adult stem cell-based models (2 studies). This trend reflects growing interest in developmentally relevant and human-relevant platforms for assessing mixture effects, particularly for endpoints related to differentiation, neurodevelopment, and reproductive or developmental toxicity. In the 3D model category, 3D spheroids (5 studies) and 3D organoids (4 studies) were more frequently employed than organ-on-a-chip platforms (1 study), indicating that structurally advanced but technically more accessible *in vitro* systems currently predominate over more complex microphysiological systems in mixture research. Exposure route-based models showed the highest overall representation, and within this category inhalation-based systems were more common (17 studies) than dermal models (11 studies). In studies designed to simulate inhalation exposure, air–liquid interface (ALI) assays were commonly used because they enable more realistic modeling of exposure to aerosols, particulate matter, volatile chemicals, and other airborne mixtures. In contrast, dermal exposure assessment mainly relied on *in vitro* and *ex vivo* skin tissue models, including reconstructed epidermis, full-thickness skin models, and *ex vivo* skin explants. These platforms are particularly suitable for evaluating mixtures relevant to cosmetics, consumer products, and environmentally derived samples in which skin contact represents a major route of exposure.

### 4.1 HTS/HCI

High-throughput screening (HTS) and high-content imaging/screening (HCI/HCS) can be understood as representative system-based screening NAMs that enable the quantitative assessment of biological responses induced by chemicals or mixtures in cell-based systems. HTS typically relies on automated high-density plate formats to process large numbers of samples simultaneously, focusing on relatively limited readouts for rapid response evaluation. In contrast, HCI/HCS integrates fluorescence imaging with computational image analysis to capture multidimensional phenotypic information, including cell morphology, organelle status, and protein localization. These differences may influence how each approach contributes to mixture toxicity assessment.

HTS-based approaches have primarily been used either for large-scale interaction screening or for profiling whole-mixture toxicity by treating mixtures as single exposure units. An automated 384-well OECD TG 455-based estrogen receptor reporter assay was established to evaluate the estrogenic activity of binary mixtures of consumer product-related endocrine-disrupting chemicals. Mixture effects were classified as synergistic, antagonistic, or additive by comparing observed responses with concentration addition predictions, and the resulting dataset was further used to train random forest (RF) and deep neural network (DNN) models (Kim et al., 2025). In contrast, BioMAP Diversity PLUS panel was used to assess cigarette smoke and next-generation tobacco aerosol samples across 12 human primary cell systems and quantified 148 protein biomarkers related to inflammation, vascular responses, tissue remodeling, and immunomodulation (Simms et al., 2021). In this case, mixtures were treated as complex exposure units, and overall toxicity profiles were compared across samples rather than resolving component-level interactions.

HCI-based approaches have been more frequently used to characterize phenotypic and mechanistic alterations induced by mixtures at the cellular level. HCI was applied to a human mesenchymal stem cell adipogenesis model and quantified adipocyte number, size, and lipid accumulation following exposure to bisphenol mixtures (Norgren et al., 2022). Cell Painting-based high-content analysis was used in MCF-10 A cells to examine PFOS/PFOA mixtures and reported potential synergistic effects on cell proliferation and morphological changes at low concentrations (Pierozan et al., 2023). Live-cell HCS was combined with deep learning-based image analysis in BEAS-2B Cells to quantify mitochondrial network disruption induced by airborne pesticide mixtures (Charrasse et al., 2023). A similar phenotypic profiling strategy was also applied to environmental chemical combinations using Cell Painting across multiple human cell lines, further illustrating the utility of untargeted morphological profiling for distinguishing concentration- and combination-dependent responses (Rietdijk et al., 2022). In some cases, HCI-based evaluation was further integrated with molecular-level analyses. For example, Cell Painting with metabolomics and lipidomics were combined in HepG2 and HepaRG cells to investigate environmentally relevant PFAS mixtures. In this study, mixtures were treated as single exposure units, and the analysis identified concentration-dependent and non-monotonic responses, including morphological alterations, mitochondrial abnormalities, and perturbations in lipid, bile acid, steroid, and amino acid metabolism (Alijagic et al., 2024). Collectively, these studies suggest that while HTS is particularly useful for rapid prioritization and large-scale interaction screening, HCI/HCS provides richer phenotypic resolution for identifying subtle, cell-type-specific, and mechanistically informative mixture responses. This makes HCI especially valuable when the objective is not only to detect mixture effects, but also to characterize how those effects manifest at the cellular and subcellular levels.

### 4.2 OMICS

Omics-based NAMs can be understood as approaches that capture molecular-level perturbations induced by mixture exposure across different biological layers, including genes, proteins, and metabolites, enabling interpretation at the pathway and network levels. These approaches extend beyond endpoint-specific measurements and allow relatively unbiased profiling of biological responses. Depending on the molecular layer analyzed, they can be classified into transcriptomics, proteomics, metabolomics, and integrated multi-omics, which may provide complementary information for interpreting complex mixture effects. While HTS/HCI focuses on scalable response profiling, Omics-based NAMs enable deeper mechanistic interpretation of mixture-induced biological changes.

Transcriptomics encompasses a range of platforms with different resolutions, including RNA-seq, microarray, TempO-Seq, and targeted transcriptomics. These approaches have been applied across diverse biological systems, including hepatocyte models, placenta, endometrium, and ovary, and have covered a wide range of chemical groups such as PFAS, metals, herbicides, pesticides, pharmaceuticals, and phthalate mixtures (Addicks et al., 2023; Carberry et al., 2025; Freedman et al., 2025; Hara-Yamamura et al., 2021; Lichtenstein et al., 2021; Tarvainen et al., 2023; Valente et al., 2025; Visser et al., 2024). Some studies directly compared gene expression responses between single compounds and mixtures, whereas others interpreted mixture responses in relation to expected additive effects, such as those based on concentration addition or relative potency factor concepts. Metabolomics has been applied to environmentally relevant complex mixtures, such as PM2.5 or nitrosamine mixtures (Rodrigues et al., 2023; Zhao et al., 2021). In these studies, the primary focus was on capturing metabolite-level changes following mixture exposure rather than quantitatively resolving interactions among individual components. Proteomics-based approaches have been used in more complex test systems, such as airway organotypic models or 3D human brain neurospheroids, and have addressed specific molecular layers, including extracellular vesicle cargo changes, protein modification, and covalent adduct formation (Casella et al., 2022; Louati et al., 2024; Vitucci et al., 2024). Integrated multi-omics approaches combine transcriptomics with metabolomics, proteomics, or both (Casella et al., 2022; Dong et al., 2023; Papaioannou et al., 2020; Yang et al., 2025), and have been primarily applied in hepatic *in vitro* systems such as HepG2 or HepaRG cells. These studies have aimed to link alterations across multiple molecular layers at the pathway or network level, rather than simply presenting parallel datasets.

### 4.3 Stem cell

Stem cell-based *in vitro* NAMs can be understood as approaches that utilize the differentiation capacity of human-derived stem cells to recapitulate specific tissues or developmental processes *in vitro*, allowing the assessment of how chemical mixtures affect these processes. These models can be categorized based on cellular origin and differentiation stage into human embryonic stem cell (hESC)-based models, human induced pluripotent stem cell (hiPSC)-based models, and adult stem cell-derived systems.

HiPSC-based systems include a wide range of lineage-specific and functionally differentiated models, such as cardiomyocytes, neural stem cells, neuron/astrocyte co-cultures, hepatocytes, endothelial cells, and multi-lineage differentiated systems (Cervetto et al., 2023; Chen, Z. et al., 2021; Davidsen et al., 2020; Pistollato et al., 2021; Walker et al., 2021). These models have been applied to diverse exposure scenarios, including tobacco extracts, POPs mixtures, binary metal mixtures such as Pb–MeHg, multicomponent environmental mixtures, and post-disaster sediment extracts. Functional endpoints assessed in these systems include synaptogenesis, neurite outgrowth, BDNF expression, and neurotransmitter release. hESC-based models were identified in only one study and were used to recapitulate early developmental processes such as embryogenesis (Li, B. et al., 2025). 23-component environmental pollutant mixture reflecting maternal exposure profiles was applied to an hESC-based early embryonic model and evaluated apoptosis, ROS generation, cell cycle arrest, autophagy, and germ layer specification. This study showed that hESC-based systems can capture mixture-induced perturbations during early developmental stages that extend beyond simple cytotoxicity. Adult stem cell-based systems have been used to evaluate adipogenic differentiation in response to mixtures (Bérubé et al., 2023; Reina-Pérez et al., 2022). Studies using human adipose-derived stem cells or mesenchymal stem cells assessed the effects of BPA/BPF/BPS mixtures or complex environmental mixtures on lipid accumulation and adipogenic programming, in relation to signaling pathways such as ER, PPARγ, RXRα, GR, and TRβ. Some studies reported non-monotonic responses or effects exceeding simple additivity.

### 4.4 3D models

Three-dimensional NAMs can be broadly classified into spheroids, organoids, and organ-on-a-chip/microphysiological systems (MPS) according to their degree of structural complexity and physiological relevance. Among the 10 studies included in this review, organoids were the most frequent category with five studies, followed by spheroids with four studies, whereas organ-on-a-chip/MPS was represented by only one study. This distribution indicates that the current application of 3D NAMs in mixture toxicity assessment is still centered mainly on spheroid- and organoid-based systems.

Spheroid-based studies were primarily used to evaluate structural alterations and tissue-level phenotypes induced by mixtures. These included 3D aggregate models derived from ovarian, endometrial, hepatic, and tumor-related systems and were used to examine representative environmental mixtures such as phthalates, bisphenols, and persistent organic pollutants (Bérubé et al.; Gogola-Mruk et al., 2021; Lavogina et al., 2024; Nikanfar et al., 2025; Reina-Pérez et al., 2022; Štampar et al., 2023). In these studies, defined mixtures based on human biomonitoring or environmental exposure data were used to assess extracellular matrix remodeling, impaired cell-cell adhesion, oxidative stress, and increased invasiveness, using 3D architecture-related readouts such as compactness, invasion area, ECM protein deposition, and tight junction assembly. Organoid-based studies were used to assess more complex developmental and functional disturbances. This category included liver organoids, cerebral organoids, brain organoids with optic vesicles, cortical brain organoids, and 3D bronchospheres exposed to diverse environmental mixture scenarios such as microplastics, PFAS, heavy metals, wastewater effluents, and complex EDC mixtures (Caporale et al., 2022; Chen, S. et al., 2023; Cheng et al., 2024; Lu et al., 2024; Niu et al., 2024). These studies extended beyond simple cytotoxicity assessment to evaluate higher-order tissue responses, including neural layer formation, synaptogenesis, optic vesicle development, mitochondrial dysfunction, mucus hypersecretion, altered lipid metabolism, and neurodegeneration-like changes. Some studies further combined organoid systems with scRNA-seq, transcriptomics, or proteomics to analyze cell type-specific responses and pathway-level perturbations. Organ-on-a-chip/MPS was limited to one study, but it represented a platform capable of incorporating exposure conditions that are difficult to reproduce in static culture systems. In this study, a microfluidic bionic lung chip was used to deliver an e-cigarette aerosol mixture consisting of propylene glycol, vegetable glycerin, and nicotine under gas–liquid interface conditions, allowing the assessment of oxidative stress, inflammation, apoptosis, and ribosomal dysfunction under more realistic inhalation-like exposure conditions (Li, Z. et al., 2024).

### 4.5 Exposure route-relevant *in vitro* model

Exposure route-based *in vitro* NAMs are not always clearly defined as an independent technological category within conventional NAM classification frameworks. However, analysis of the mixture toxicity studies included in this review revealed a recurring pattern in which *in vitro* or *ex vivo* test systems designed to simulate specific exposure routes were repeatedly used to better reflect real-world exposure scenarios. Rather than merely increasing cellular or structural complexity, these approaches aim to enhance the realism of mixture exposure assessment by incorporating into the experimental system the physical delivery mode and contact environment through which chemicals are encountered in the human body. Such route-of-exposure-relevant approaches have developed primarily around pulmonary and dermal exposure, as these tissues represent major contact sites for environmental and consumer-related mixtures. This tendency is particularly notable in mixture toxicity research, where reproducing realistic combined exposure scenarios is often more important than in single-chemical studies. In this context, these models highlight the value of test systems that reflect exposure delivery processes more faithfully than conventional submerged culture conditions.

Pulmonary exposure models are primarily based on air–liquid interface (ALI) systems and have been used to evaluate a wide range of inhalation-relevant mixtures, including ambient air pollution, vehicle exhaust, combustion emissions, woodsmoke, e-cigarette aerosols, volatile organic compounds, urban particulate matter, 3D printer emissions, black carbon, gunshot fumes, atmospherically aged particles, and PAH mixtures (Abzhanova et al., 2024; Ashrin et al., 2025; Binder et al., 2022; Chang et al., 2021; Colvin et al., 2024; Dilger et al., 2023; Fang et al., 2023; Gualtieri et al., 2024; Hakkarainen et al., 2022; Henriquez et al., 2025; Jaber et al., 2025; Mariussen et al., 2021; Ruth et al., 2023; Sayyed et al., 2022; Vojtisek-Lom et al., 2024; Zimmermann et al., 2025). Test systems range from conventional cell lines such as A549, BEAS-2B, and Calu-3 to more advanced models including hAELVi, primary bronchial epithelial tissues, EpiAirway™, and MucilAir. Frequently assessed endpoints include cytotoxicity, ROS generation, pro-inflammatory cytokine release, barrier dysfunction, DNA damage, and transcriptomic changes. In some cases, these models were also used to compare distinct emission sources or exposure fractions within the same scenario, thereby allowing more refined interpretation of mixture effects. For example, gunshot fume studies comparing different ammunition types demonstrated differential cytotoxic and genotoxic responses under direct ALI exposure conditions (Mariussen et al., 2021), while systems toxicology studies of wood combustion aerosols showed that the gas phase, particularly carbonyl compounds, could dominate transcriptomic and genotoxic responses despite the concurrent presence of particulate matter (Dilger et al., 2023). These examples further illustrate that ALI-based models can capture not only overall inhalation hazard, but also phase-specific or source-dependent contributions within complex airborne mixtures.

Dermal exposure models are centered on reconstructed epidermis, full-thickness skin models, and *ex vivo* skin explants (Carlsson et al., 2024; De Souza et al., 2024; Mizumachi et al., 2020; Monnot et al., 2021; Page et al., 2023; Pambianchi et al., 2021; Patatian et al., 2021; Sahli et al., 2022; Wedekind et al., 2023). These systems have been used to evaluate mixtures derived from consumer products and environmental sources, including surfactants, oxidized fragrance mixtures, phthalate UVCBs, textile-derived leachates, urban pollution, and combined exposure scenarios such as diesel exhaust with cosmetic formulations. Endpoints include tissue viability, IL-1α and IL-18 release, CD54 expression, T Cell responses, histological changes, barrier integrity, oxidative damage, and DNA methylation. In some studies, artificial sweat extraction was used to generate textile-derived mixtures, which were then evaluated using downstream bioassays such as KeratinoSens™. Collectively, these dermal models extend beyond simple topical hazard screening by incorporating barrier function, migration processes, and tissue-level modulation, thereby providing a more realistic framework for assessing skin-relevant mixture exposures.

## 5 Potential role of NAMs in mixture assessment

**FIGURE 5:**
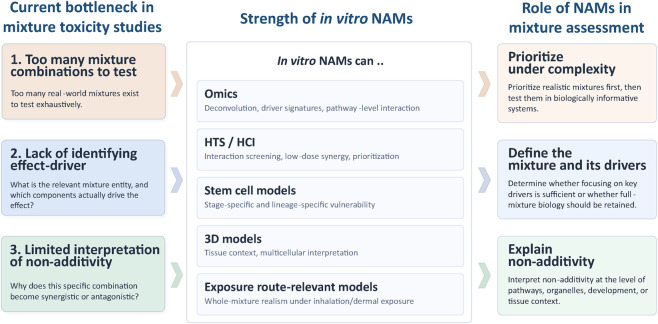
Overview of major bottlenecks in mixture toxicity assessment and the contributions of different *in vitro* NAMs.

Despite longstanding progress in mixture toxicology, the assessment of chemical mixtures remains constrained by several challenges that are intrinsic to mixtures themselves rather than to chemical hazard assessment in general. Real-life co-exposures are often incompletely characterized, chemically variable, and compositionally dynamic, making it difficult to define the relevant mixture entity and to determine whether observed effects can be sufficiently represented by a few dominant components (Tkalec et al., 2024). In addition, mixtures may produce non-additive responses that cannot be readily explained by conventional additivity-based frameworks, particularly when interactions emerge in a pathway-, organelle-, or developmental stage-specific manner (Hirano et al., 2023). Finally, the sheer diversity of real-world co-exposures makes comprehensive testing impractical, such that mixture assessment increasingly depends on prioritization, tiered evaluation, and the integration of multiple evidence streams (OECD, 2020). In this context, the role of NAMs in mixture assessment lies not merely in replacing conventional assays, but in addressing these mixture-specific challenges through more mechanistically informative, biologically relevant, and strategically deployable testing frameworks (Sewell et al., 2024). Figure 5 illustrates how different *in vitro* NAMs can help address key bottlenecks in mixture assessment.

### 5.1 High-throughput screening of mixture combinations under combinatorial complexity

A third challenge is that real-world co-exposures are too numerous, heterogeneous, and dynamic to be comprehensively assessed using fixed-composition strategies. Reviews in regulatory and environmental mixture science consistently note that humans and ecosystems are simultaneously exposed to large numbers of chemicals through multiple exposure routes, such that mixture assessment depends less on exhaustive testing than on prioritization, tiered evaluation, and the integration of exposure, hazard, and bioactivity evidence (Bopp et al., 2019).

The studies included in this review illustrate how NAMs can operationalize such a strategy. Exposure-route-relevant platforms, particularly ALI systems, enabled direct assessment of ambient air, photochemically aged soot, and complex aerosol systems while preserving real exposure conditions rather than reducing them to oversimplified component-based designs (Chang et al., 2021; Colvin et al., 2024; Hakkarainen et al., 2022). At the same time, advanced *in vitro* systems such as spheroids, organoids, reconstructed tissues, and *ex vivo* models supported biologically meaningful interpretation of selected complex or reconstructed mixtures by retaining tissue architecture, barrier properties, and multicellular organization. For instance, Lu et al. used a 3D cerebral organoid model to show that PFAS mixtures induced cell type-specific neurotoxicity and AD-like neuropathology (Lu et al., 2024), whereas Cheng et al. demonstrated in liver organoids that biologically digested micro/nanoplastic mixtures caused mitochondrial dysfunction and reductive stress (Cheng et al., 2024). Niu et al. further showed in a 3D bronchosphere model that complex wastewater mixtures induced barrier disruption, inflammation, and mucus hypersecretion (Niu et al., 2024). These examples suggest that NAMs do not overcome combinatorial complexity by testing every possible mixture, but rather by enabling a more realistic and tiered framework in which exposure-relevant mixtures are prioritized and subsequently evaluated in biologically informative systems capable of supporting mechanistic interpretation.

### 5.2 Identification of effect-driving components

A fundamental challenge in mixture assessment is that real-life co-exposures are often incompletely characterized, variable across time and space, or composed of poorly defined substances, making it difficult to determine whether a component-based or whole-mixture strategy is more appropriate and which chemicals are actually driving the observed effect (Escher et al., 2020). Thus, mixture assessment involves not only estimating combined toxicity, but also defining the relevant mixture entity and deconvoluting its toxicity drivers through integrated chemical, biological, and statistical evidence.

The studies reviewed here indicate that Omics-based NAMs are particularly useful in addressing this question because they can resolve mixture responses into patterns shared with individual components and additional perturbations that emerge only after exposure to the full mixture. For example (Yang et al., 2025), showed that BaP accounted for part of the hepatotoxic response induced by a food-relevant PAH4 mixture, while the full mixture still elicited additional transcriptomic and metabolomic perturbations that could not be reproduced by the single compound alone. Similarly (Carberry et al., 2025), identified PFOS as the major driver within a ternary PFAS mixture in HepG2 cells, while transcriptomic benchmark modeling confirmed the biological relevance of the mixture itself. Together, these findings suggest that NAMs support mixture assessment by clarifying whether a dominant component can serve as an adequate proxy or whether the full mixture induces distinct biology that should be retained in assessment.

### 5.3 Explaining why specific combinations produce non-additive effects

A second major challenge is that conventional mixture assessment may detect deviations from additivity, yet often fails to explain why a given combination becomes synergistic or antagonistic (Bopp et al., 2019). Recent literature emphasizes that non-additivity may arise through multiple mechanisms, including pathway convergence, toxicokinetic interactions, competition between biological responses, or susceptibility differences associated with developmental stage and biological context. Accordingly, the key question is not simply whether a mixture departs from additivity, but at which biological level and by what mechanism that departure occurs.

The reviewed NAM studies provide several examples of how this issue can be addressed mechanistically (Dong et al., 2023). showed through integrated transcriptomics and metabolomics that the same DBP/DEHP mixture acted synergistically on purine and pyrimidine metabolism while acting antagonistically on fatty acid metabolism, indicating that non-additivity may be pathway-dependent rather than a fixed property of the mixture as a whole (Pierozan et al., 2023). further demonstrated that low-dose PFOS/PFOA co-exposure induced synergistic proliferative effects accompanied by marked morphological alterations associated with PXR and Akt/β-catenin signaling, whereas (Charrasse et al., 2023) showed that trace co-occurring pesticides potentiated mitochondrial toxicity by lowering toxicity thresholds and intensifying disruption of mitochondrial architecture. Stem cell-based studies extended this interpretation to developmental susceptibility (Wang et al., 2023). showed that melamine and cyanuric acid synergistically inhibited hepatocyte differentiation at concentrations where the individual chemicals had little effect, with ROS generation identified as a key mechanistic driver. Likewise, Davidsen et al. found that a human-relevant POP mixture was largely additive overall, yet produced endpoint-specific synergy or antagonism across neurodevelopmental endpoints, with synaptogenesis emerging as a particularly sensitive target. Collectively, these studies show that NAMs allow non-additivity to be interpreted not simply as a deviation from prediction models, but as a biologically localized phenomenon occurring at the level of pathways, organelles, developmental programs, or specific functional endpoints.

## 6 Perspectives

A key future challenge in chemical mixture toxicology is not simply to generate more data, but to determine what kind of evidence is most informative for different mixture assessment contexts. As the field moves beyond single-substance paradigms, the role of advanced *in vitro* NAMs should not be viewed only in terms of replacing conventional assays or increasing experimental complexity. Rather, their more important contribution may lie in improving how evidence is generated, interpreted, and integrated for mixtures. One important direction is to move away from treating all mixtures as if they require the same assessment logic. In practice, mixtures differ substantially in how they should be approached: some are relatively well defined and suitable for component-based comparison, whereas others are poorly characterized, variable over time, or better understood as whole exposure entities (More et al., 2021).

Future progress will therefore depend on aligning the experimental strategy with the nature of the mixture itself. In this context, advanced *in vitro* NAMs can serve as flexible tools for selecting the appropriate level of resolution, from component-specific mechanistic analysis to whole-mixture response profiling. A second priority is to strengthen the biological interpretability of mixture effects. One persistent limitation in mixture toxicology is that non-additive responses are often reported descriptively, without a clear understanding of where in the biological system those deviations emerge. Advanced *in vitro* NAMs offer an opportunity to address this limitation by linking mixture responses to specific levels of biological organization, such as molecular pathways, organelle stress responses, cellular states, tissue architecture, or developmental programs.

Future studies should therefore focus less on whether a mixture is simply additive or non-additive, and more on identifying the biological context in which interaction becomes meaningful. Another important perspective is the need to build fit-for-purpose testing frameworks rather than search for a single universally optimal NAM platform. The diversity of advanced *in vitro* systems reviewed in this field suggests that no individual model can address all mixture-related questions. High-content or Omics-based systems may be particularly useful for mechanistic signal detection, whereas stem cell-based, 3D, and route-of-exposure-relevant models may be better suited for understanding biological susceptibility, tissue context, and realistic exposure conditions. The future value of NAMs in mixture assessment will therefore depend on how effectively these systems are positioned within tiered and question-driven workflows. From a regulatory perspective, the most meaningful contribution of advanced *in vitro* NAMs may be their ability to support decision-relevant interpretation rather than merely produce biologically sophisticated data. For broader regulatory uptake, future research should place greater emphasis on reproducibility, transferability, comparability with existing approaches, and clarity of use context (Malinowska and Whelan, 2025; Ouedraogo et al., 2025). This means that studies should be designed not only to demonstrate biological novelty, but also to clarify what specific assessment question a given NAM can answer more effectively than conventional methods.

**FIGURE 6:**
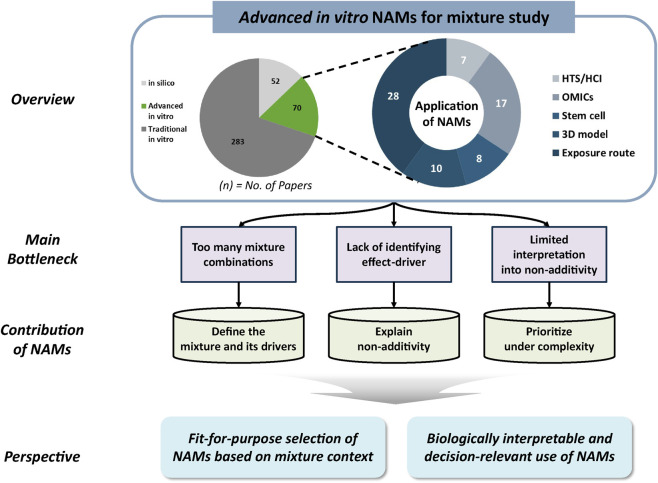
Overview of current applications, key bottlenecks, and future perspectives of advanced *in vitro* NAMs in chemical mixture toxicity assessment

Ultimately, future progress in mixture toxicity assessment will depend on whether advanced *in vitro* NAMs can be incorporated into clearly defined, question-driven testing strategies. Rather than valuing NAMs primarily for their technical novelty, the field should increasingly evaluate them according to how effectively they support mixture-specific decision needs, including mixture definition, interaction interpretation, driver identification, and prioritization. From this perspective, the next stage of development will require not only continued platform innovation, but also stronger alignment between biological model choice, analytical purpose, and regulatory use context (Figure 6).
